# Supplementary material for: Targeting of Immune Cells by Human Adenoviruses: CD46 Receptor Density Influences Entry of Chimaeric Ad5F35 into Natural Killer Cells
Source: Int J Mol Sci. 2026 Oct 16;27(20):9183. doi: 10.3390/ijms27209183 (PMC13705385; doi:10.3390/ijms27209183)
Supplement: Supplementary file 1 [file ijms-27-09183-s001.zip › ijms-4538116-supplementary.pdf]

Supplementary Figures, Tables and Legends:

Barr et al: Targeting of immune cells by human adenoviruses – CD46 receptor density influences entry of chimaeric Ad5F35 into natural killer cells

Supplementary Figure S1:

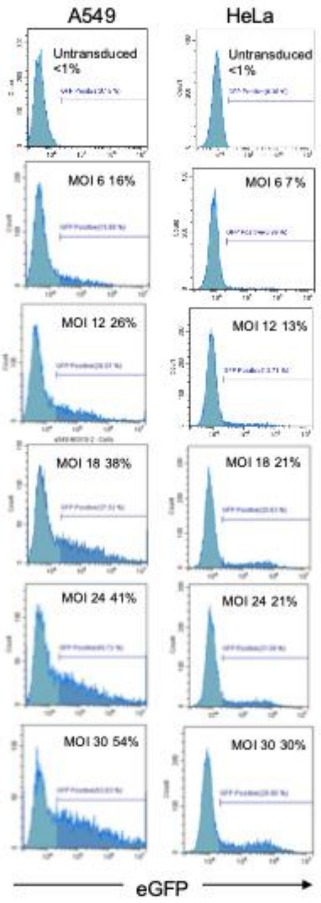

Transduction of A549 cells and HeLa cells with increasing MOI of Ad5F35-GFP.

Transduction of A549 cells and HeLa cells with Ad5F35-EGFP across a range of MOIs. Transduction was determined by EGFP expression 24 hrs post-transduction. Percentage of cells transduced at each MOI is indicated.

Supplementary Figure S2:

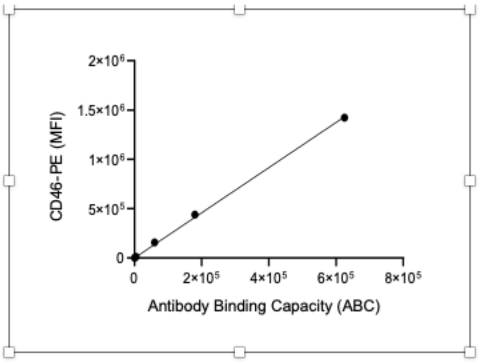

Example standard curve for interpolation of antibody binding capacity (ABC).

The standard curve was constructed from ABC of beads shown in Figure 4A using software provided by the manufacturer.

Supplementary Tables:

Supplementary Tables 1 and 2 provided as separate Excel files

Supplementary Table S1:

Dataset for Figures 1A and 1B. The 889 human cell types and tissues analysed by the FANTOM consortium [25] are listed from A-Z in column A with expression values for the six indicated genes shown in columns B-G. Expression values are coloured in each column according to whether the expression value is between 10% and 90% of the maximum expression level in that column. The data is based on capped analyses of gene expression (CAGE) which quantitates expression from multiple start sites for each gene. Expression values shown here are total expression in each sample. This was calculated by adding expression values obtained from each start site (promoter p1, p2 etc) annotated in the FANTOM dataset. Data was downloaded from; https://fantom.gsc.riken.jp/5/sstar/Main_Page

Supplementary Table s2:

Extended version of Figure 1C showing adenovirus entry receptors and marker molecules and whether they were detected (blue) or not (white) in the cell types indicated. Source data is from the Cell Surface Protein Atlas [32], available at http://wlab.ethz.ch/cspa.
